# Novel and Known *DHX37* Variants in 46,XY DSD: Expanding the Genotypic and Phenotypic Spectrum

**DOI:** 10.3390/genes17070829

**Published:** 2026-07-21

**Authors:** Xiaocha Xu, Xiaocheng Wu, Shuai Chen, Haixia Miao, Kexin Fang, Dingwen Wu, Yi Zhang, Xin Yang

**Affiliations:** 1 Department of Genetics and Metabolism, Children’s Hospital, Zhejiang University School of Medicine, National Clinical Research Center for Children and Adolescents’Health and Disease, Hangzhou 310051, China; 2 Zhejiang Key Laboratory of Neonatal Diseases, Hangzhou 310052, China; 3 Binjiang Institute of Zhejiang University, Hangzhou 310053, China

**Keywords:** *DHX37* gene, 46,XY DSD, ribosome biogenesis, domain, whole-exome sequencing

## Abstract

Objective: To investigate the clinical characteristics, inheritance patterns, and genotype–phenotype correlations of DHX37 variants in 46,XY disorders of sex development (DSD). Methods: We retrospectively reviewed 108 patients with 46,XY karyotype who underwent DSD evaluation and trio-based whole-exome sequencing (trio-WES) at our center between January 2021 and December 2025. Six probands with DHX37 variants and no concurrent pathogenic or likely pathogenic variants in other known DSD-associated genes were analyzed in detail. Clinical presentations, endocrine profiles, imaging findings, and pedigree data were collected. Variant segregation was confirmed by Sanger sequencing; variants were assessed using in silico prediction, conservation analysis, and structural modeling, and were classified according to ACMG/AMP guidelines. Results: The six probands exhibited marked phenotypic heterogeneity, with manifestations ranging from complete gonadal dysgenesis to mild testicular underdevelopment with gynecomastia. Six heterozygous *DHX37* missense variants were identified across distinct functional domains: one in RecA1 [c.1000C>T (p.Arg334Trp)], four in RecA2 [c.1379T>C (p.Val460Ala), c.1432G>A (p.Gly478Arg), c.1730A>G (p.Asp577Gly), and c.1954G>A (p.Val652Ile)], and one in the linker region proximal to the HA2 domain [c.2180C>T (p.Thr727Met)]. Of these, p.Arg334Trp is an established pathogenic variant; p.Gly478Arg has been reported previously, albeit in the same patient included in the present study; and the remaining four were novel. According to ACMG/AMP guidelines, p.Arg334Trp was classified as pathogenic, and the remaining five as variants of uncertain significance. Conclusions: This study provides additional case evidence supporting the pathogenicity of p.Arg334Trp and expands the DHX37 variant spectrum. Computational and structural analyses suggest that p.Gly478Arg may underlie the testicular regression syndrome phenotype of the corresponding proband and that the four novel variants may be involved in testicular development. However, these genotype–phenotype correlations remain speculative; larger cohorts and in vitro functional assays are warranted to confirm these associations.

## 1. Introduction

*DHX37* (DEAH-box helicase 37) encodes an RNA helicase abundantly expressed in the central nervous system throughout embryonic development and adulthood [1], as well as in fibroblasts, endothelial cells, epididymal epithelial cells, Leydig cells, and germ cells at various maturation stages [2]. RNA helicases constitute a large group of enzyme superfamilies (SF1–SF6) that utilize ATP hydrolysis to modulate RNA metabolism and play pivotal roles in transcription, splicing, translation, and ribosome biogenesis [3].

*DHX37* variants have been linked to two distinct disorders: 46,XY sex reversal 11 (OMIM#273250) and neurodevelopmental disorder with brain anomalies and with or without vertebral or cardiac anomalies (OMIM#618731). Two mechanisms have been proposed: (i) defective ribosome biogenesis: DHX37 promotes U3 snoRNA dissociation from pre-ribosomal particles via its RNA-dependent ATPase activity, a step critical for 5′ ETS cleavage and 40S subunit maturation [4,5,6]. Dysfunction leads to 30S pre-rRNA accumulation and impaired 21S-to-18S-E conversion, which reduces mature 40S production and mRNA translation efficiency, with tissue-specific consequences including testicular dysgenesis and neuronal and synaptic dysfunction [7]; and (ii) nucleolar stress and p53 activation: impaired ribosome biogenesis disrupts nucleolar integrity and drives the nucleoplasmic translocation of nucleolar proteins such as NPM1 and ARF. This translocation inhibits MDM2-mediated p53 degradation; the resulting p53 stabilization ultimately induces cell cycle arrest or apoptosis [8].

*DHX37* is a key gene associated with gonadal dysgenesis in 46,XY DSD, a spectrum that encompasses complete gonadal dysgenesis (CGD), partial gonadal dysgenesis (PGD), testicular regression syndrome (TRS), and 46,XY ovotesticular DSD [9]. Notably, *DHX37* variants have been detected in up to 25% of TRS and 16% of PGD cohorts [10,11], and the underlying mechanism has been characterized as a “nucleolar stress–WNT/β-catenin activation–p53-dependent apoptosis” cascade [12,13,14]. Although prior studies have begun to delineate the clinical heterogeneity of *DHX37* variants across functional domains, systematic genotype–phenotype analyses remain limited. Building on this foundation, the present study reports novel *DHX37* variants that further broaden the known variant spectrum. We used an integrative framework combining structural modeling, evolutionary conservation analysis, and in silico pathogenicity prediction to explore potential domain-specific correlations with clinical phenotypes. These findings complement the existing evidence base and may inform future functional validation and molecular diagnostic strategies for *DHX37*-related 46,XY DSD.

## 2. Materials and Methods

### 2.1. Subjects

This study retrospectively reviewed patients with a 46,XY karyotype who were evaluated for suspected DSD at the Children’s Hospital, Zhejiang University School of Medicine between January 2021 and December 2025. Of 108 patients who underwent trio-based whole-exome sequencing (trio-WES), six individuals carrying variants in the *DHX37* gene without additional pathogenic or likely pathogenic variants in other DSD-associated genes were selected for further analysis. Clinical presentations were heterogeneous and included hypospadias, micropenis, cryptorchidism, gynecomastia, and ambiguous genitalia. Written informed consent was obtained from the guardians of all enrolled patients. This study was approved by the Institutional Ethics Committee (No. 2024-IRB-0147-P-01).

### 2.2. Clinical and Endocrine Evaluation

Comprehensive clinical, laboratory, and genetic data were collected, including age at presentation, sex assigned at birth, and physical examination findings. External genitalia and secondary sexual characteristics were evaluated by experienced specialists. Breast development and pubic hair growth were staged according to Tanner criteria. Pelvic ultrasonography was performed by senior operators. Serum hormone levels (FSH, LH, testosterone, and others) were measured using the Abbott Alinity analyzer in a single laboratory to ensure consistency.

### 2.3. Molecular Studies

Genomic DNA was extracted from peripheral blood and subjected to whole-exome sequencing (WES). Exome capture was performed using the KAPA HyperExome V2 probe kit (Roche Molecular Systems, Inc., Pleasanton, CA, USA). Sequencing libraries were prepared for paired-end 150 bp reads and sequenced on the DNBSEQ-T7 platform (MGI Tech., Beijing, China), with a mean target coverage of ≥100× and >95% of targets covered at ≥20×. High-quality reads were aligned to the GRCh38/hg38 reference genome using BWA-MEM (v0.7.17). Duplicate reads were removed, and base quality scores were recalibrated prior to single-nucleotide variant (SNV) and insertion/deletion (indel) calling using GATK HaplotypeCaller (v4.4.0.0). Rigorous quality control was applied at three levels. At the sample level, metrics included estimated contamination, chimera rate, insert size distribution, transition-to-transversion (Ti/Tv) ratio, and heterozygous-to-homozygous call ratio. At the variant level, filtering incorporated standard GATK annotations (QUAL, QD, MQ, FS, SOR, DP, GQ, MQRankSum, ReadPosRankSum). At the batch level, QC was performed to detect technical artifacts, with samples or variants that failed their respective thresholds excluded from downstream analysis. To filter common polymorphisms, variants with minor allele frequency (MAF) ≥ 0.1% in gnomAD v3.1.2 (genomes) and the 1000 Genomes Project were removed unless annotated as pathogenic or likely pathogenic in ClinVar. More stringent thresholds (MAF < 0.01%) were applied to known autosomal-dominant DSD-associated genes. Candidate variants in known DSD-associated genes were validated by Sanger sequencing, and segregation analysis was performed in available family members. In silico protein damage prediction was conducted using SIFT, PolyPhen-2, MutationTaster, REVEL, and CADD. All variants were classified according to ACMG/AMP 2015 guidelines, with 2022 updates applied where relevant.

### 2.4. Copy Number Variation (CNV) Analysis

CNV calling from WES data was performed using ExomeDepth (v1.1.15) and cn.MOPS (v1.38.0), with only concordant calls retained for downstream analysis. Quality control criteria included the following: (i) mean target coverage ≥ 100×, with >95% of targets at ≥20×; (ii) Pearson’s correlation coefficient > 0.97 between the sample and pooled reference set; (iii) statistical significance threshold of Bayes Factor ≥ 10 (ExomeDepth) or posterior probability ≥ 0.95 (cn.MOPS); and (iv) O/E read depth ratio ≤ 0.7 for deletions and ≥1.3 for duplications. Only CNVs spanning ≥ 3 consecutive exons were retained, given the reduced reliability of smaller events from WES data. CNVs were filtered against population databases, including gnomAD v2.1.1 (exomes) and v3.1.2 (genomes) and the 1000 Genomes Project using a frequency threshold of <0.1% for duplications and <0.05% for deletions, unless annotated as pathogenic or likely pathogenic in ClinVar. Pathogenicity classification followed ACMG/ClinGen (2019 and 2023) criteria for copy number variant interpretation, incorporating ClinGen dosage sensitivity scores and DECIPHER phenotypic associations. Candidate CNVs were validated by quantitative PCR (qPCR) for intragenic events and chromosomal microarray analysis (CMA) for larger rearrangements. Segregation analysis was performed in available family members.

### 2.5. Structural Modeling

Domain and sequence analysis. Domain annotations of DHX37 were obtained from InterPro and UniProt databases. Homologous sequences from ten species were retrieved from the NCBI database, and multiple sequence alignment was performed using Clustal Omega V1.2.4.

Homology modeling and structural analysis. The three-dimensional structure of human DHX37 was predicted by I-TASSER using the murine Dhx37 crystal structure (PDB: 6O16_A, 2.88 Å) as the template. Variant modeling and structure visualization were carried out using PyMOL V3.1.8.

### 2.6. DSD-Associated Genes and Exclusion Criteria

A consensus DSD-associated gene set was assembled from established gene panels (Eggers et al., 2016 [15]; Genomics England PanelApp v3.0) that cover genes involved in testis determination, androgen synthesis and action, and AMH signaling. Variants were filtered in three tiers: (i) common polymorphisms were excluded based on population allele frequency thresholds (MAF ≥ 0.1% in gnomAD and the 1000 Genomes Project; ≥0.01% for X-linked variants); (ii) synonymous or deep intronic variants (>50 bp from splice junctions) were retained only when predicted to disrupt splicing using MaxEntScan or SpliceAI; and (iii) variants classified as benign or likely benign according to ACMG/AMP 2015 guidelines (with 2022 updates applied) were excluded. Priority was given to rare variants within conserved functional domains (PhastCons ≥ 0.9), namely those classified as pathogenic, likely pathogenic, or variants of uncertain significance (VUS) with consistent in silico evidence (predicted to be damaging by ≥2 independent tools or CADD score ≥ 20). If no causal variant was identified in the primary panel, extended candidate genes implicated in gonadal development were screened using identical filtering criteria. The possibility of oligogenic inheritance was assessed by evaluating the co-occurrence of rare, damaging variants across all screened DSD-associated genes. All prioritized variants were validated by Sanger sequencing, and family-based segregation analysis was performed to evaluate inheritance consistency.

## 3. Results

### 3.1. Clinical Characteristics

The six study participants exhibited marked phenotypic heterogeneity. Five individuals met diagnostic criteria for 46,XY DSD. Patient 6 presented with complete gonadal dysgenesis (CGD), manifesting as female external genitalia, female sex assignment, and dysgenetic gonads located within the inguinal canals. Laparoscopy revealed absent Müllerian structures. Patient 3 demonstrated testicular regression syndrome (TRS), with bilateral cryptorchidism, micropenis, elevated basal FSH (25.5 IU/L) and inappropriately normal LH (1.06 IU/L), and absent testosterone response to hCG stimulation (0.47 nmol/L following 1500 IU/m^2^ every other day for three doses; reference > 7.0), consistent with primary testicular failure and Leydig cell dysfunction. Patient 1 presented with bilateral cryptorchidism and micropenis. Although clinical features were highly suggestive of TRS, definitive subtyping was precluded by the absence of histopathological confirmation. Patients 2 and 5 exhibited milder phenotypes: Patient 2 presented with prepubertal gynecomastia and mild testicular dysgenesis; Patient 5 had isolated micropenis with bilateral high scrotal testes. Neither case was definitively classified. Patient 4 did not meet diagnostic criteria for 46,XY DSD and presented with isolated gynecomastia and an otherwise normal testicular examination. Detailed clinical, endocrine, and imaging findings for each individual are summarized in Table 1.

### 3.2. Molecular Data

Six heterozygous missense variants in the *DHX37* gene were detected in six patients, with no concurrent pathogenic variants or copy-number abnormalities in other DSD-associated genes. These variants, which spanned distinct functional domains, were distributed as follows. One variant was located in the RecA1 domain: c.1000C>T (p.Arg334Trp), positioned between motifs Ia and Ib. Four variants were located in the RecA2 domain: c.1379T>C (p.Val460Ala) at the RecA1–RecA2 interdomain interface, proximal to motif IV; c.1432G>A (p.Gly478Arg) within motif IV (DEAH); c.1730A>G (p.Asp577Gly) in the central intermotif region between motifs IV and V; and c.1954G>A (p.Val652Ile) in the C-terminal segment of RecA2, adjacent to the HA2 domain and positioned between motifs V and VI. One variant was located in the linker region proximal to the N-terminus of the HA2 domain: c.2180C>T (p.Thr727Met). Of these, p.Arg334Trp was a previously established pathogenic variant; p.Gly478Arg had been documented in the literature, though only in the same patient as reported herein; and the remaining four were novel, with no prior documentation in public databases. According to ACMG/AMP guidelines, p.Arg334Trp was classified as pathogenic, whereas the remaining five were VUS. In silico predictions of protein-damaging effects and their pathogenicity classifications are summarized in Table 2.

**Figure 1:**
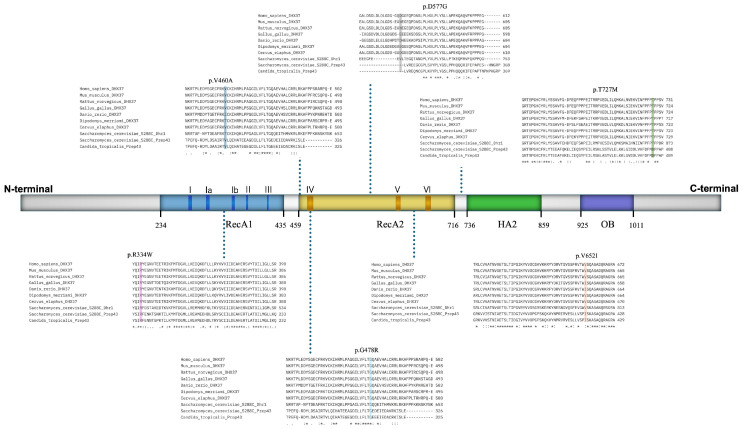
Domain diagram and evolutionary conservation of *DHX37*. The schematic shows the domain structure of *DHX37* and the multiple sequence alignment of variant sites across eight species. RecA1 (residues 234–435, blue) mediates ATP binding/hydrolysis and primary RNA interaction; RecA2 (459–716, yellow) is responsible for RNA binding and unwinding; HA2 (736–859, green) is the helicase-associated domain 2; OB (925–1011, purple) is the oligonucleotide/oligosaccharide-binding domain. Six variant sites (p.R334W, p.G478R, p.V460A, p.D577G, p.V652I, p.T727M) are labeled. The DHX37 domain information data is sourced from the InterPro (https://www.ebi.ac.uk/interpro/protein/UniProt/Q8IY37/), accessed on 20 April 2026. Asterisks (*), colons (:), and dots (.) indicate identical, conserved, and semi-conserved residues, respectively. Motif nomenclature follows the classic DEAH-box helicase motifs I–VI as described by Boneberg et al., 2019 [17].

**Figure 2:**
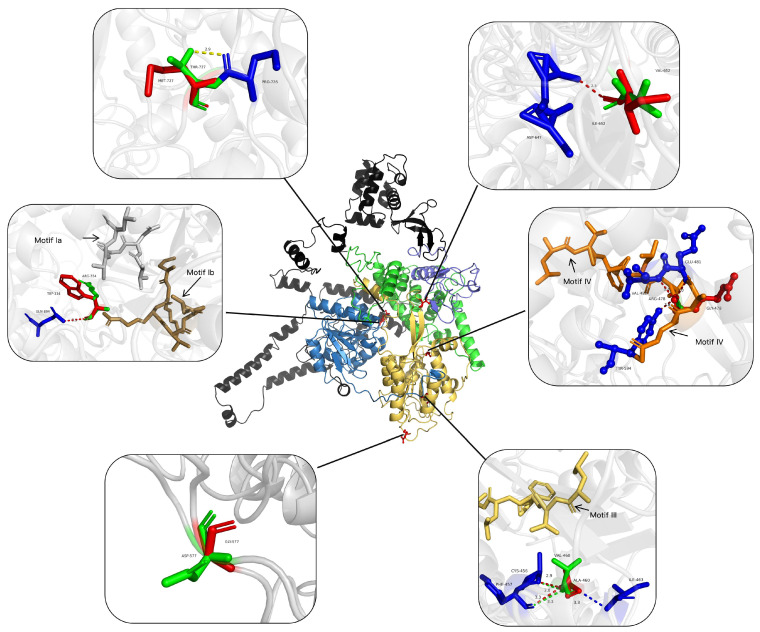
Three-dimensional structure prediction of wild-type DHX37 protein and local conformational. analysis of variant sites. The three-dimensional predicted structure of the full-length human wild-type DHX37 protein is displayed in cartoon mode. The N-terminal and C-terminal regions are shown in black, the RecA1 domain in blue, the RecA2 domain in yellow, the HA2 domain in green, and the OB domain in light purple. The six magnified sub-panels display the local structural microenvironment of each variant site: green ball-and-stick models represent wild-type amino acid residues, red ball-and-stick models represent mutated amino acid residues, and blue ball-and-stick models represent adjacent amino acid residues that form hydrogen bonds with the target site, intuitively illustrating the potential impact of variants on local amino acid residue interactions and domain conformations. The presented DHX37 protein structure is a predicted homology model.

Evolutionary conservation of the six variants is summarized in Figure 1. p.Arg334Trp and p.Gly478Arg exhibit complete amino acid conservation across all examined orthologs, indicating that these positions are under stringent purifying selection and subject to critical structural constraints. Deviation from the wild-type residue at these positions may impair function. In contrast, p.Val460Ala, p.Asp577Gly, p.Val652Ile, and p.Thr727Met exhibit incomplete conservation, with interspecies amino acid variability indicating reduced selective constraint and greater evolutionary flexibility. This suggests greater tolerance to amino acid substitution at these positions. This dichotomy supports provisional stratification: positions with complete cross-species identity are less tolerant of substitution, whereas those with documented variability are more permissive. Structural modeling of the six variants is presented in Figure 2. Evolutionary conservation and structural modeling alone are insufficient to establish pathogenicity; functional studies and additional clinical cases are needed for validation.

### 3.3. Inheritance Pattern

**Figure 3:**
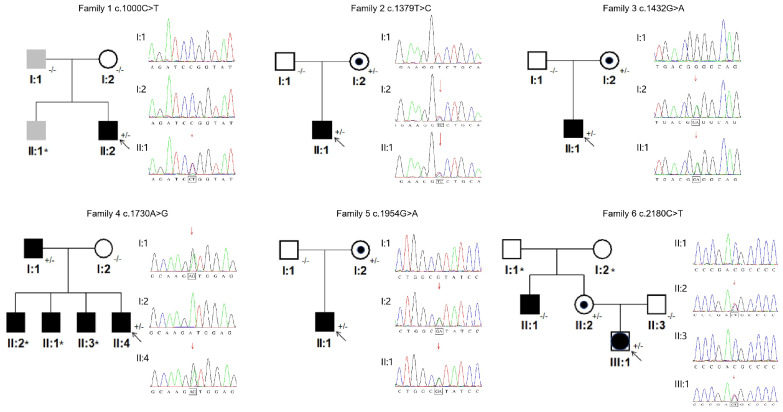
Pedigrees and representative chromatograms illustrating DHX37 variants in 46,XY DSD. The genotype of available family members is shown. Squares represent males and circles represent females. Solid squares represent affected 46,XY subjects raised as boys, whereas squares containing solid circles represent affected 46,XY subjects raised as girls. Symbols containing a black dot represent carriers of the variant. The gray square indicates a man with unilateral testicular agenesis. +/− indicates heterozygous mutation, −/− indicates the wild-type allele, * indicates subjects not tested for the mutation, black arrow represents the proband, and red arrow represents the mutation site.

Pedigree analysis supported a sex-limited autosomal-dominant inheritance pattern: one variant occurred *de novo*, four were maternally inherited, and one was paternally inherited. All 46,XY carriers exhibited abnormal phenotypes, whereas 46,XX carriers were asymptomatic (Figure 3).

## 4. Discussion

The DHX37 protein (NP_116045, 1157 amino acids) contains four core functional domains: RecA1 (ATP binding and hydrolysis), RecA2 (forms an RNA-binding channel with RecA1), HA2 (3′-end RNA anchoring), and OB-fold (5′-end RNA binding), as well as a characteristic C-terminal domain (CTD) that serves as the UTP14A-binding region mediating ATPase activation [17,18]. Since the initial report by da Silva et al. in 2019 [2], more than 60 patients with 46,XY DSD associated with *DHX37* variants have been reported worldwide. Over 25 distinct variants have been identified, predominantly clustered within the RecA1 and RecA2 domains, with the majority being missense variants; among these, p.R308Q (36.67%) and p.R674W (11.67%) represent the most recurrent mutational hotspots [19,20]. Literature-reported *DHX37* pathogenic variants demonstrate marked domain-specific pathogenicity and considerable phenotypic heterogeneity. Variants affecting the RecA1 domain are associated with the most severe and comprehensive phenotypic spectrum of DSD, whereas variants within the RecA2 domain constitute the most prevalent DSD-associated mutational region, with phenotypes relatively restricted to micropenis and cryptorchidism. By contrast, variants localized to the HA2 and OB domains exhibit a comparatively attenuated association with classic DSD phenotypes and are more frequently implicated in spermatogenic failure, implying that these structural domains likely exert more specialized, temporally restricted functions during later stages of gonadal development and gametogenesis [19].

Among the five 46,XY DSD patients enrolled in this study, only Patient 1 carried a variant located within the RecA1 domain (c.1000C>T, p.Arg334Trp) and presented with hypergonadotropic hypogonadism and severe testicular dysgenesis. This variant has been reported previously in two cases (1 CGD and 1 TRS) [10,16], and our findings further substantiate its clinical pathogenicity. In silico protein damage prediction tools classified this variant as deleterious. Structural modeling revealed its localization within a highly conserved region of the RNA-binding groove in the RecA1 domain, adjacent to motifs Ia/Ib. The amino acid substitution alters a conserved residue in this region, which may influence helicase function. However, given the absence of functional data, these observations remain speculative, and confirmation of pathogenicity requires further experimental evidence and additional case reports. The inheritance pattern in this family was complex. Paternal relatives exhibited apparent paternal clustering of similar phenotypes, although with considerable phenotypic heterogeneity. The father had a history of unilateral cryptorchidism with spontaneous descent, whereas the elder brother presented with right-sided cryptorchidism that was surgically corrected. Trio-WES analysis of the father did not identify any definitive pathogenic variants in known DSD-associated genes; however, the elder brother did not undergo genetic testing or targeted variant validation. Sanger sequencing demonstrated the variant as *de novo* in the proband, with both parents harboring the wild-type allele. The phenotypic clustering among paternal relatives raised two plausible explanations: (i) confined gonadal mosaicism in the father with paternal cryptorchidism representing an unrelated incidental finding; or (ii) combined somatic-germline mosaicism in the father, with a somatic mosaic fraction below the detection limit of Sanger sequencing. Because germline or sperm testing in the father and genetic confirmation in the brother were not undertaken, and multi-tissue mosaicism analysis was not performed, the precise inheritance pattern could not be definitively established. Consequently, the variant lacked meaningful segregation evidence. Nevertheless, in clinical practice, paternal gonadal mosaicism should be considered and appropriately evaluated in such scenarios.

Four other patients carried distinct missense variants within the RecA2 domain, comprising three with 46,XY DSD and one with minor anomalies of sex development. These included c.1379T>C (p.Val460Ala) in Patient 2, c.1432G>A (p.Gly478Arg) in Patient 3 (the same case previously reported in the literature [13]), c.1730A>G (p.Asp577Gly) in Patient 4, and c.1954G>A (p.Val652Ile) in Patient 5. All four variants were classified as VUS. Among the three patients with 46,XY DSD, Patient 3 exhibited the most severe phenotype, presenting with testicular regression syndrome (TRS); Patient 2 manifested concealed micropenis with bilateral cryptorchidism; and Patient 5 demonstrated a relatively milder phenotype, characterized by bilaterally palpable but high-riding testes, a short penile shaft with the majority buried subcutaneously (corporal spongiosal development was relatively preserved), a narrow preputial orifice precluding glans exposure, and well-developed scrotum. Patient 4 presented solely with discordance between serum hormone levels and testicular volume development, accompanied by gynecomastia. Conservation analysis and in silico protein damage prediction tools yielded partially concordant yet partially discordant results across these four variants. The p.Gly478Arg substitution was predicted as deleterious by multiple algorithms, consistent with its localization within a highly conserved region. By contrast, p.Val460Ala, p.Asp577Gly, and p.Val652Ile exhibited poor evolutionary conservation; among them, p.Val460Ala and p.Val652Ile received conflicting predictions—some tools classifying them as tolerated and others as deleterious—whereas p.Asp577Gly yielded relatively concordant results, with most algorithms indicating low pathogenicity. Structural modeling revealed that p.Gly478Arg resides within Motif IV (DEAH), the core catalytic engine of ATP-dependent RNA unwinding [17]. p.Val460Ala, p.Asp577Gly, and p.Val652Ile all localize to regions of relatively low structural constraint—situated at domain junctions or motif linkers—and may only subtly alter protein function rather than completely disrupting core enzymatic activity. Notably, the *DHX37* variants documented in previously reported oligogenic DSD cases, which co-occurred with pathogenic variants in *NR5A1* [11], *MAMLD1* [21], or *SOX9* [22], also mapped to non-catalytic regions. This raises the possibility that variants in these regions may function as phenotypic modifiers rather than fully penetrant monogenic disease drivers. However, we did not identify any additional pathogenic or likely pathogenic variants in other known DSD-associated genes within our study cohort. Oligogenic inheritance thus remains an intriguing hypothesis for *DHX37*-related disorders, yet the present dataset provides no supportive evidence for this model. Further functional assays and expanded patient cohorts are necessary to validate this hypothetical genetic interaction.

Additionally, we identified a c.2180C>T (p.Thr727Met) variant in the N-terminal linker region adjacent to the HA2 domain in a patient (Patient 6) with complete gonadal dysgenesis (CGD). The HA2 domain is known to cooperate with RecA1 and RecA2 in stabilizing RNA substrate binding and regulating the unwinding conformation [17]. Variants in the HA2 domain and its flanking regions remain rarely reported. Peng et al. noted that variants in the HA2 and OB domains were predominantly identified in cohorts with primary spermatogenic failure, whereas their contribution to classic DSD phenotypes remains less clearly established [19]. For instance, two variants in the HA-proximal region or HA domain—c.2177dup (p.Thr727fs) and c.2209G>A (p.Ala737Thr)—were associated with infertility and cryptorchidism, respectively [19]. The severe CGD phenotype observed in Patient 6 appears discordant with these limited reports. Conservation analysis indicated that Thr727 is a moderately conserved residue; in silico protein damage prediction yielded discordant results, with the majority of algorithms classifying the substitution as deleterious and a minority classifying it as tolerated. Structural modeling predicted a potentially altered local conformation in the HA2 linker region, but the functional consequences of this substitution remain undefined. Whether such structural changes extend beyond spermatogenic maintenance to influence earlier testicular determination processes remains purely speculative. The hypothesis that the biological importance of HA2 domain integrity in gonadal development has been underestimated remains unproven. The inheritance pattern in this family further complicates phenotypic interpretation. The patient’s maternal uncle (46,XY) had dysgenetic testes (subsequently excised); however, Sanger sequencing confirmed the absence of the p.Thr727Met allele in this relative, indicating a lack of co-segregation. The uncle did not undergo systematic genetic evaluation, and the etiology of his testicular dysgenesis remains undetermined. Whether additional gonadal development-related genetic factors in this family may synergize with the *DHX37* variant, or whether the uncle’s condition represents an independent pathological event, remains indeterminate. In summary, existing clinical and molecular evidence regarding variants in the HA2 domain and its adjacent regions remains extremely limited. The precise functional role of this domain in gonadal development and the pathogenicity assessment of variants therein remain poorly defined at the mechanistic level. The causal relationship between p.Thr727Met and the CGD phenotype observed in Patient 6 should be interpreted with considerable caution, and establishment of its pathogenicity awaits in vitro functional assays, model organism studies, and additional clinical cases with rigorous genotype–phenotype correlation.

Patients 2 and 4 presented with gynecomastia. Previous studies have proposed that *DHX37* may indirectly preserve the microenvironment for sex hormone synthesis by maintaining testicular structural integrity; consequently, pathogenic *DHX37* variants may disrupt the androgen-to-estrogen ratio [14]. However, given Patient 4’s age at the time of genetic testing, physiological pubertal gynecomastia should also be considered in the differential diagnosis. Family history revealed that the father and three older brothers of Patient 4 had varying degrees of gynecomastia; however, this family was lost to follow-up, precluding further clinical evaluation, and the genetic status of the three brothers remains unknown. Therefore, the correlation between the proband’s clinical phenotype, the family history of gynecomastia, and the p.Asp577Gly variant cannot be established based on the available data. Both Patients 4 and 5 had comorbid attention-deficit/hyperactivity disorder (ADHD), which partially overlaps with the neurodevelopmental phenotypic spectrum reported in association with *DHX37* variants. Whether *DHX37* variants exhibit pleiotropic effects and whether they might disrupt neuronal proteostasis by interfering with ribosome biogenesis remain to be determined [7,19]. Alternatively, this comorbidity may reflect independent genetic or environmental factors unrelated to the *DHX37* genotype.

In conclusion, we report a 46,XY DSD patient harboring the recurrent variant c.1000C>T (p.Arg334Trp), consistent with previous reports. Additionally, through in silico prediction of protein damage, evolutionary conservation analysis, and structural modeling, we identified five novel missense variants—c.1379T>C (p.Val460Ala), c.1432G>A (p.Gly478Arg), c.1730A>G (p.Asp577Gly), c.1954G>A (p.Val652Ile), and c.2180C>T (p.Thr727Met)—thereby expanding the *DHX37* mutational spectrum. The clinical phenotypes observed in carriers of these novel variants, ranging from testicular regression syndrome to partial gonadal dysgenesis and minor anomalies of sex development, do not permit definitive genotype–phenotype correlations; any inferred patterns are purely conjectural, may reflect ascertainment bias or coincidental clustering, and await functional validation in larger cohorts.

## Figures and Tables

**Table 1:** Clinical characteristics of the probands and their available family members.

Characteristic	Patient 1	Patient 2	Patient 3	Patient 4	Patient 5	Patient 6
Date of Birth	2021/11/7	2012/11/7	2017/6/27	2010/11/25	2018/1/7	2021/2/7
Age at Presentation/Age at Hormonal Testing	Neonatal/7M	7Y10M/7Y11M	Neonatal/4Y	Unknown/13Y4M	2Y/7Y6M	Neonatal/1Y
Social Sex	Male	Male	Male	Male	Male	Female
Karyotype	46,XY	46,XY	46,XY	46,XY	46,XY	46,XY
Clinical diagnosis	NOS	NOS	TRS	Minor sex developmental anomaly *	NOS	CGD
Gonadal Palpability	Bilateral cryptorchidism	Bilateral palpable testes, 1 mL	Bilateral cryptorchidism	Bilateral palpable testes, Tanner G4	Bilateral palpable testes	Scrotum empty, no testes palpable
Phenotype	Micropenis, bilateral empty scrotum, non-palpable testes	Bilateral breast development with nodules and tenderness, advanced bone age, suspected pituitary lesion	Micropenis, small preputial orifice	Breast development; sleep disorders, ADHD	Micropenis, small preputial orifice; ADHD	Female external genitalia
External Genitalia	Micropenis	Micropenis with buried appearance	Micropenis, small preputial orifice	PH Tanner Stage 5	Micropenis	Ambiguous external genitalia, PH Tanner Stage 1
Imaging Findings (Scrotal/ testicular/ epididymal ultrasound/ Breast)	Epididymis-like echoes bilaterally in the inguinal region; hyperechoic lesions suggestive of testicular atrophy	No obvious abnormality in bilateral testes/ Epididymis; Breast echoes detected bilaterally. Left epididymal cyst	Gonad detected in the left inguinal region, ovary possible; gonad detected at right internal ring, testis possible; right hydrocele	No obvious abnormality; Breast echoes detected bilaterally	Bilateral testes in a high position	Hypoechoic areas at bilateral inguinal canal internal rings, dysgenetic gonads possible; Uterus and bilateral ovaries not detected, vagina approximately 1.8 cm, lower segment shared orifice with urethra
Family History	Father had unilateral cryptorchidism with spontaneous descent; elder brother had right cryptorchidism	N	N	Family history of male gynecomastia	N	Maternal uncle had testicular dysgenesis (surgically removed)
FSH (IU/L) (Ref)	79.63 (<0.1–2.5)	4.95 (<0.1–2.5)	25.5 (<0.1–2.5)	6.13 (1.5–10.0)	1.3 (<0.1–2.5)	115.24 (<0.1–2.5)
LH (IU/L) (Ref)	12.92 (<0.1–1.5)	2.4 (<0.1–2.0)	1.06 (<0.1–2.0)	3.62 (2.0–12.0)	0.05 (<0.1–2.0)	13.57 (<0.1–1.5)
T(nmol/L) (Ref)	0.62 (<0.7)	1.13 (<0.7)	<0.69 (<0.7)	11.57 (10.0–25.0)	<0.45 (<0.7)	0.47 (<0.7)
Serum AMH Level (ng/mL) [Age at Examination]	1.24 [7M]	13.43 [10Y2M]	0.28 [4Y]	Not measured	Not measured	0.14 [1Y7M]
Sex Assignment (Reassigned to Male/Continued Male)	Continued male	Continued male	Continued male	Continued male	Continued male	Continued female
Müllerian Derivatives on Imaging Studies or Laparoscopy	Not detected	Not detected	Not detected	Not detected	Not detected	Vagina visible on ultrasound, not detected on laparoscopy
DHX37 variant, zygosity, inheritance, Detection time	c.1000C>T (p.Arg334Trp), het, de novo, 2021.7	c.1379T>C (p.Val460Ala), het, Maternal, 2022.6	c.1432G>A (p.Gly478Arg), het, Maternal, 2022.10	c.1730A>G (p.Asp577Gly), het, Paternal, 2022.10	c.1954G>A (p.Val652Ile), het, Maternal, 2024.4	c.2180C>T (p.Thr727Met), het, Maternal, 2025.11

Reference range refers to normal laboratory reference intervals according to age and sex. FSH, follicle stimulating hormone; LH, luteinizing hormone; T, testosterone; AMH, anti-Müllerian hormone; DSD, disorders/differences in sex development; CGD, complete gonadal dysgenesis; PGD, Partial Gonadal Dysgenesis; TRS, testicular regression syndrome; NOS: Not otherwise specified; Y: years; M: month; PH, pubic hair; N: No other affected family members identified. Het: heterozygous mutation. * P4 presented with isolated gynecomastia and otherwise normal testicular examination, without meeting diagnostic criteria for 46,XY DSD according to the Chicago consensus, this individual was included to explore the phenotypic spectrum of DHX37 variants beyond classic DSD presentations.

**Table 2:** *DHX37* variants identified in 46,XY DSD patients of this study.

Patient	Nucleotide Change	Amino Acid Alteration	Zygosity	Functional Domain	Motif	GnomAD MAF	GnomAD MAF (East Asian)	In Silico Prediction Tools					ACMG Classification	Inheritance	Reported Status	Phenotype
								SIFT	PolyPhen-2	MutationTaster	CADD	REVEL				
1	c.1000C>T	p.Arg334Trp	Het	RecA1	/	-	-	Deleterious	Probably damaging	Disease_causing	12.79	0.309	P (PS3+PM1+PM2+PP3+PP5)	De novo	Reported [10,16]	PGD
2	c.1379T>C	p.Val460Ala	Het	RecA2	/	-	-	Tolerated	Benign	Polymorphism [probably harmless]	23.1	0.358	VUS (PM1+PM2+PP4)	Maternal	Novel	Breast development, Concealed penis
3	c.1432G>A	p.Gly478Arg	Het	RecA2	IV	-	-	Deleterious	Probably damaging	Disease_causing	29.8	0.013	VUS (PM1+PM2+PP4)	Maternal	Reported ^[a]^	TRS
4	c.1730A>G	p.Asp577Gly	Het	RecA2	/	5/246838	5/18294	Tolerated	Benign	Polymorphism [probably harmless]	11.77	0.349	VUS (PM1+PP3+PP4)	Paternal	Novel	Breast development; ADHD
5	c.1954G>A	p.Val652Ile	Het	RecA2	/	10/282822	0/19950	Tolerated	Probably damaging	Disease_causing	25.2	0.064	VUS (PM1+PM2+PP4)	Maternal	Novel	Micropenis; ADHD
6	c.2180C>T	p.Thr727Met	Het	/	/	5/69898	3/7120	Deleterious	Probably damaging	Disease_causing	24.6	0.595	VUS (PM1+PM2+PP4)	Maternal	Novel	CGD

Het: heterozygous mutation. P, pathogenic; LP, likely pathogenic; VUS, variant of uncertain significance. a. c.1432G>A has been reported earlier [13] and is the same patient as in this study.

## Data Availability

The data generated in this study can be found within the article. Raw data are available from the corresponding author on request.
